# Incontinentia Pigmenti Misdiagnosed as Neonatal Herpes Simplex Virus Infection

**DOI:** 10.1155/2018/1376910

**Published:** 2018-06-13

**Authors:** Fahimeh Abdollahimajd, Minoo Fallahi, Mohammad Kazemian, Yalda Nilipour, Mitra Radfar, Sedigheh Tahereh Tehranchi

**Affiliations:** ^1^Skin Research Center, Shahid Beheshti University of Medical Sciences, Tehran, Iran; ^2^Neonatal Health Research Center, Research Institute for Children Health, Shahid Beheshti University of Medical Sciences, Tehran, Iran; ^3^Pediatric Pathology Center, Research Institute for Children Health, Shahid Beheshti University of Medical Sciences, Tehran, Iran

## Abstract

Incontinentia pigmenti (IP) is an X-linked dominant neurocutaneous syndrome with ophthalmologic, neurologic, cutaneous, and dental manifestations and in most cases antenatally lethal in boys. Occasionally, typical IP may occur in boys due to Klinefelter syndrome or a genomic mosaicism. Skin lesions are observed in 4 stages: blistering, verrucous linear plaques, swirling macular hyperpigmentation, followed by linear hypopigmentation that develop during adolescence and early adulthood. Neonatal herpes simplex virus (HSV) infection can be manifested in 3 forms: localized, disseminated, and central nervous system (CNS) involvement. Timely diagnosis and treatment of neonatal HSV infection is critical. In this case report, we present a 12-day female newborn with a history of maternal genital HSV in second trimester and vesicular lesions on the upper and lower limbs that was appeared at first hours of life. She was admitted in the maternity hospital that was born and was treated by antibiotic and acyclovir for 11 days. Then, she readmitted for her distributed vesicular lesions. The results of blood and CSF for HSV PCR were negative. Eventually the diagnosis for incontinentia pigmenti was made by consultation with a dermatologist, and skin biopsy confirmed the diagnosis.

## 1. Introduction

Incontinentia pigmenti (IP) is a rare X-linked dominant neurocutaneous syndrome with a prevalence of 0.7/100,000 live birth and ophthalmologic, neurologic, cutaneous, and dental manifestations [1, 2]. The first cases of the disease were reported by Garrod in 1906 [3], after that Bloch and Sulzberger defined this disorder in 1926 and 1928, respectively [4], as a clinical syndrome with a constellation of unique features that include typical cutaneous manifestations. Up until 1987, only 700 cases had been reported in the literature but have a worldwide distribution and more common among white patients.

The disease is probably underreported because many mild or uncomplicated cases are likely unrecognized.

Abnormalities of the tissues and organs in this disease are derived from the ectoderm and neuroectoderm; actually, it is a kind of ectodermal dysplasia.

Involvement of the skin, teeth, nails, and hair is seen in conjunction with neurologic and ophthalmologic anomalies [5].

It is mostly a male lethal syndrome and more than 95% of reported cases of IP occur in females, and it rarely occurs in males with Klinefelter syndrome [6].

Transmission of the disease in females by a lionization results in functional mosaicism of X-linked genes. In most patients, vesicular stages of IP are present at birth or develop in the first few weeks of life. Characteristic stages are blistering (from birth to about four months of age), verrucous plaques (for several months), swirling macular hyperpigmentation (from about six months and persist during childhood and usually fade by adolescence), followed by linear hypopigmentation that develops during adolescence and early adulthood and persists indefinitely [7].

Hair, nail, and dental anomalies often manifest during infancy, and permanent neurologic (Cognitive delays/mental retardation) and ophthalmologic sequels often manifest during early infancy so that patients have retinal vascular abnormalities predisposing to retinal detachment in early childhood, Strabismus and cataract are occasionally seen. Breast anomalies, skeletal, and structural anomalies sometimes are seen in patients [8].

Neonatal HSV can be manifested in 3 types: localized, disseminated, and central nervous system (CNS) involvement [9]. Transmission of the virus from mother to fetus before the birth can be transplacental or during delivery from vaginal canals [10]. Maternal first involvement is more important than repeated infection for the transition to a fetus. Delay in diagnosis and treatment in neonatal periods can be dangerous and can accompany with a high rate of mortality and long-term complications; for this reasons, in neonates with skin manifestations or ill neonates without any recognized cause, over diagnosis and treatment by acyclovir for neonatal HSV infection may be justified.

In this case report, we describe a newborn patient suspected of HSV infection with a final diagnosis of IP.

## 2. Case Presentation

**Figure 1:**
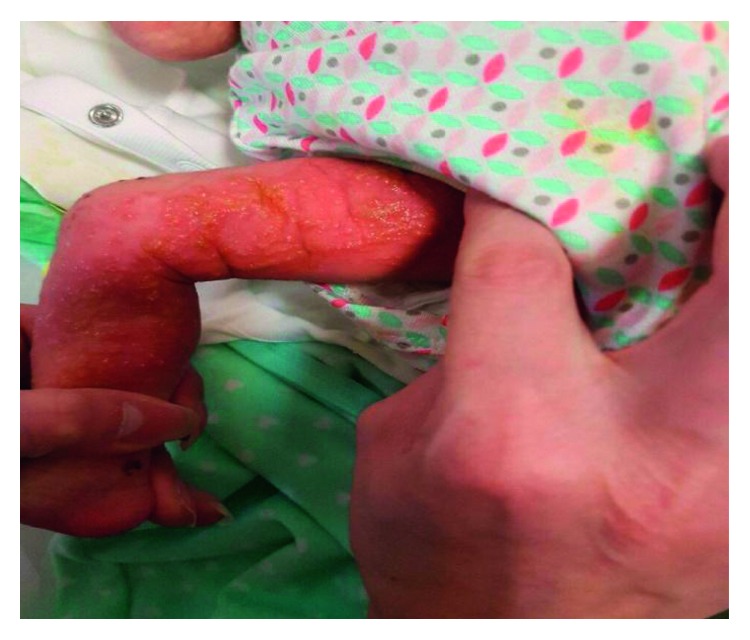
The appearance of skin lesions on the lower extremity of the patient. Note the linear pattern of the skin lesions.

**Figure 2:**
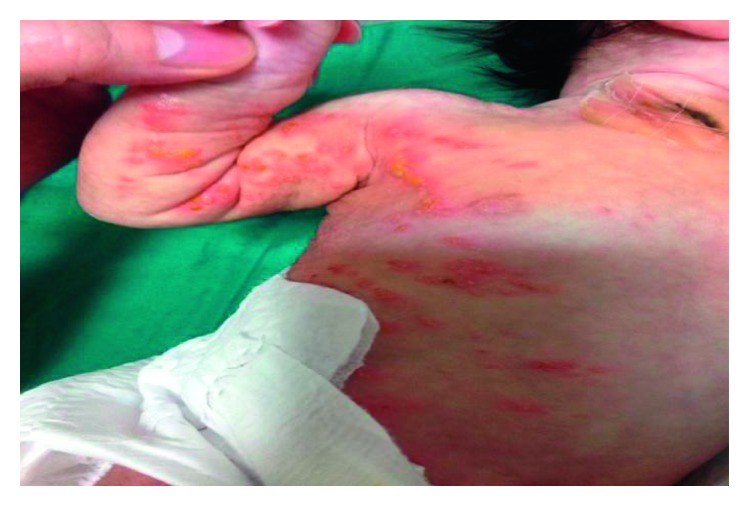
The appearance of skin lesions on the trunk and upper extremity of the patient.

**Figure 3:**
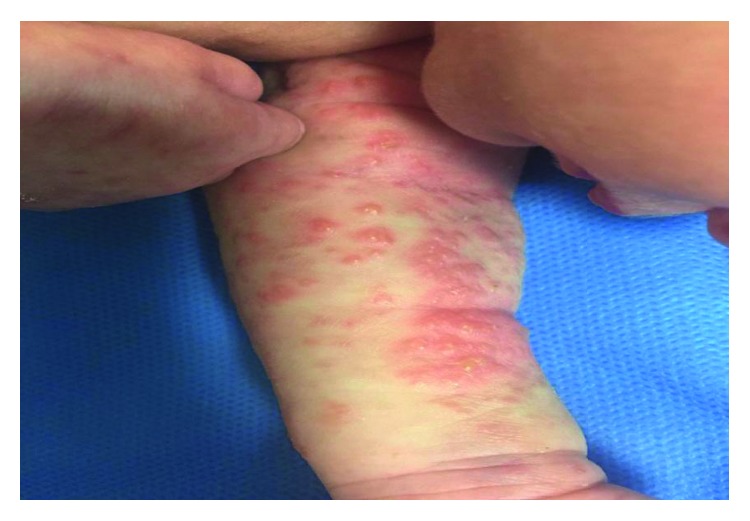
The appearance of skin lesions on the arm of the patient.

A late preterm female newborn with birth weight of 2400 grams was delivered in gestational age of 36 weeks + 6 days by normal vaginal delivery (NVD) in a private hospital, from a gravid 1, para1 mother with a past history of herpetic lesions on genitalia at the second trimester of this pregnancy that was completely treated locally with antiherpetic ointments without any lesions in vagina at the time of delivery. The newborn was admitted to the neonatal ward with a diagnosis of congenital herpes simplex infection due to vesicular lesions on the upper and lower limbs that were appeared at first hours of life. During this hospitalization, sepsis workup was done and systemic acyclovir and antibiotic therapy against staphylococcal infection was ordered. PCR evaluation of CSF and blood for herpes simplex type 1 and 2 was negative, and despite relative (not completely) improvement in cutaneous lesions, the patient was discharged from the hospital at the age of 11 days. The next day, the newborn was readmitted to our teaching pediatric hospital for her distributed vesicular lesions. PCR for re-evaluation of herpes simplex virus failed to detect it. Based on the linear pattern of skin vesicular lesions, the diagnosis of IP was raised in consultation with a dermatologist (Figures 1–3).

**Figure 4:**
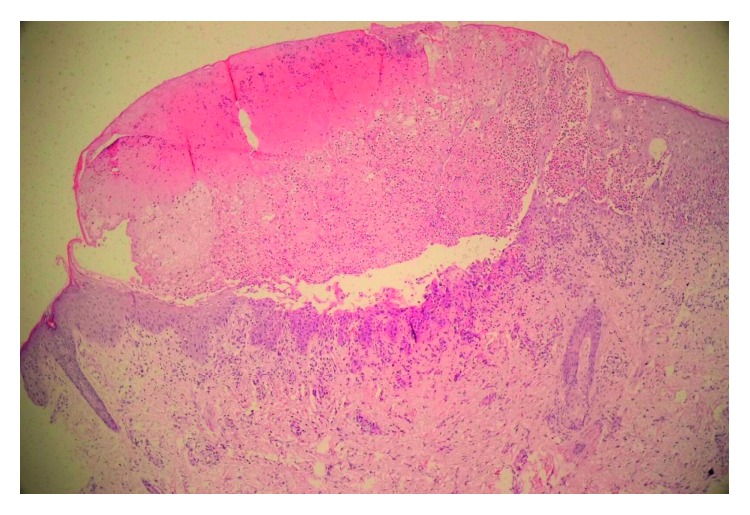
An ulcerative lesion with a fibrinous cap and intraepidermal vesicles (H&E ×40).

**Figure 5:**
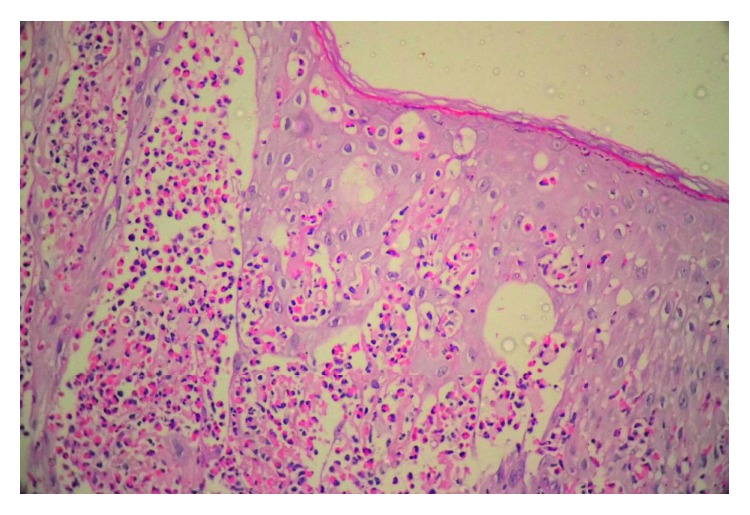
Dyskeratosis with prominent eosinophilic spongiosis and microabscess formation (H&E ×400).

Skin punch biopsy revealed an ulcerative lesion with a fibrinous cap and spongiotic vesicles (Figure 4) with marked eosinophilic exocytosis associated with dyskeratotic keratinocytes and eosinophilic intraepidermal microabscess formation (Figure 5). The aforementioned histopathologic findings confirmed the diagnosis of IP.

**Figure 6:**
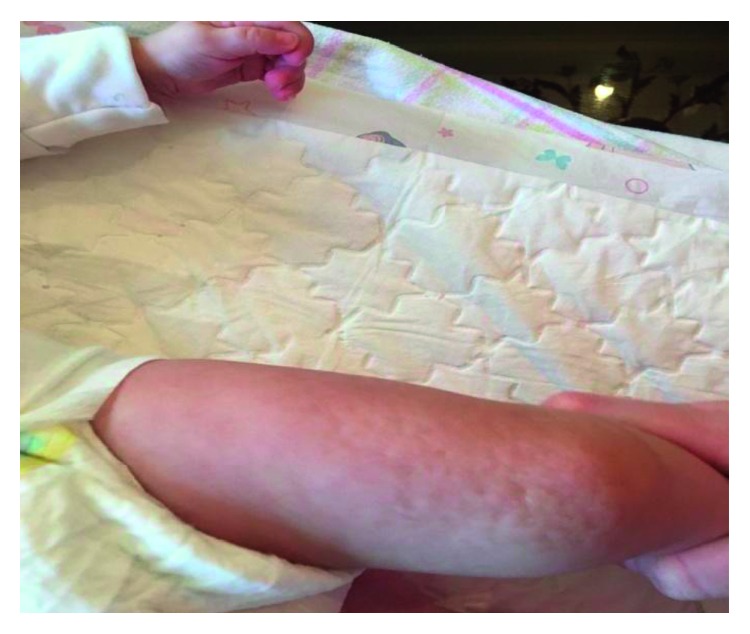
Disappearance of skin lesions in 11-month old.

Eye, CNS, and skeletal examination did not have any problem. She was prescribed topical antiseptics and mupirocin ointment and was eventually discharged with a fairly complete improvement of the lesions. In the repeated patient's follow-up until the age of 11 months, eye and CNS evaluations were unremarkable; also skin lesions have been completely improved (Figure 6).

## 3. Discussion

In this report, a case of a rare skin disease was presented, with the manifestation in the first day of life. There are some infectious disease such as varicella or HSV infection in neonatal periods, with a presentation from the early life, which timely diagnosis is critical for implication of the appropriate treatment and prevention of complications.

In this case report, the prenatal history of genital herpes simplex infection of mother was a concerned problem in neonate, because HSV infection in neonates is potentially fatal or accompanied by CNS complications and early treatment with systemic acyclovir and continuing the oral treatment up to 6 months of life can make a better prognosis with lower rate of complications. On the other hand, continuity of the skin lesions in our patient despite the proper treatment with acyclovir and a negative results of blood and CSF PCR suggested that a skin disorder is the main problem instead of HSV infection, and a consultation with a dermatologist and skin biopsy confirmed the diagnosis.

IP is an X-linked disorder involving mainly female neonate. The first manifestations appear in the early days of life as progressive to four stages: vesicular, verrucous, and hyperpigmented, and hypopigmented lesions. Other clinical features such as abnormalities of the teeth, eyes, hair, CNS, bone structures, and immune system can be observed in some patients [11].

In a reported case by Li et al. [12], a 19-day female infant diagnosed with IP was admitted due to seizure and linear erythematous and vesicular skin eruptions on the upper and lower extremities from the age of 12 days that all laboratory findings were normal except an abnormal electroencephalography (EEG) and skin biopsy which confirmed the diagnosis of IP. Although extra cutaneous manifestations of IP are presented after the neonatal period, this case developed seizure during neonatal periods; however, our patient had no other disorders except skin involvement.

Although IP is mostly a male lethal syndrome, it rarely occurs in males with Klinefelter syndrome or a genomic mosaicism. Gupta et al. [13] reported two male neonates with IP. The first case was a 5-year-old male child with hyperpigmented skin lesions started from the age of 7 days as vesicular lesions on the lower extremities and gradually spread on to the trunk, and hypodontia with peg-shaped teeth and mental retardation were also developed. The second one was a 20-day boy with vesiculobullous eruptions on both lower limbs.

Unfortunately, we did not have the precise incidence of disease in our country Iran, but due to the high rate of consanguinity, it is anticipated that some cases are missed and underdiagnosed.

Finally, in a newborn with a vesicular eruption and a history of genital HSV infection in the mother during pregnancy, it is important to consider and treat the life-threatening conditions at first.

## Data Availability

The data used to support the findings of this study are included within the article.
